# Real-World Multimodal Machine Learning for Risk Enrichment Across the Alzheimer’s Disease Spectrum

**DOI:** 10.3390/jcm15062250

**Published:** 2026-03-16

**Authors:** Nazlı Gamze Bülbül, İnci Meliha Baytaş, Efekan Kavalcı, Elvan Karasu, Başak Ceren Okcu Korkmaz, Buse Gül Belen, İsmail Serhat Musaoğlu, Ayşe Rana Övüt, Nefise Eda Arslanoğlu, Muammer Urhan, Hakan Mutlu, Mehmet Fatih Özdağ

**Affiliations:** 1 Department of Neurology, University of Health Sciences Sultan Abdulhamid Han Research and Training Hospital, Istanbul 34668, Turkeybusegulb@gmail.com (B.G.B.); ozdagf@yahoo.com (M.F.Ö.); 2 Department of Computer Engineering, Bogazici University, Istanbul 34342, Turkey; inci.baytas@bogazici.edu.tr (İ.M.B.);; 3 Department of Radiology, University of Health Sciences Sultan Abdulhamid Han Research and Training Hospital, Istanbul 34668, Turkey; i.s.musaoglu@gmail.com (İ.S.M.); hakanmutlu1@gmail.com (H.M.); 4 Department of Nuclear Medicine, University of Health Sciences Sultan Abdulhamid Han Research and Training Hospital, Istanbul 34668, Turkey; ranahoral@gmail.com (A.R.Ö.);; 5 Department of Psychology, University of Health Sciences Sultan Abdulhamid Han Research and Training Hospital, Istanbul 34668, Turkey

**Keywords:** Alzheimer’s disease, mild cognitive impairment, risk enrichment, real-world data, multimodal machine learning, volumetric MRI, FDG-PET

## Abstract

**Background and Objectives:** Mild cognitive impairment (MCI) is heterogeneous within the Alzheimer’s disease (AD) continuum, and categorical labels may not reflect biological variability. We evaluated whether multimodal machine learning using routine clinical data and neuroimaging could support biologically informed enrichment across MCI and AD in a real-world memory clinic cohort. **Methods:** We analyzed 474 patients (1547 visits) with clinical and cognitive measures, laboratory parameters, MRI regional volumes, and FDG-PET regional uptake. Elastic Net and gradient boosting models were trained using nested cross-validation with strict patient-level separation. **Results:** Model discrimination improved as additional data modalities were added, and FDG-PET contributed the largest performance improvement. Hypometabolism in posterior default mode network regions consistently emerged as the most influential predictor. In the MCI subgroup, AD-like scores showed a continuous distribution consistent with biological enrichment. **Conclusions:** Multimodal models may provide an interpretable enrichment framework in heterogeneous memory clinic populations.

## 1. Introduction

The most prevalent form of dementia worldwide, Alzheimer’s disease (AD), is a major and quickly expanding public health concern. More than 55 million people worldwide live with dementia, with Alzheimer’s disease accounting for the majority of cases. Over the next few decades, prevalence is expected to rise dramatically as the population ages [1]. AD has a major clinical and social impact on healthcare systems worldwide, caregiver well-being, and cognitive independence. As a result, both clinical practice and therapeutic research now place a high premium on early detection of people who are at high risk of illness development. Throughout the course of Alzheimer’s disease, accurate risk stratification may facilitate early intervention, increase the effectiveness of clinical trials, and support more accurate patient selection [2,3].

Mild cognitive impairment (MCI) is becoming more widely acknowledged as a heterogeneous illness and clinical stage along the Alzheimer’s disease continuum, with various trajectories [4]. Even while MCI is often seen as a prodromal stage of Alzheimer’s disease, there is significant variation in the pace and likelihood of conversion, and not all MCI patients develop dementia [5]. The chance of developing AD dementia has been demonstrated to be influenced by clinical subtypes, baseline cognitive profiles, and underlying biological traits [6,7]. Neuroimaging and biomarker studies further demonstrate that patients within the same clinical category may exhibit markedly different patterns of neurodegeneration and disease evolution [8,9]. These findings show that MCI is not a single, homogeneous clinical entity but rather a physiologically diverse state, underscoring the significance of analytical techniques that may capture this diversity [10,11].

Alzheimer’s disease is increasingly understood from a scientific standpoint as a multifactorial proteinopathy with interacting pathogenic processes rather than a single, isolated mechanism [12]. Amyloid-β accumulation, tau pathology, synaptic dysfunction, and extensive network disruption impacting cognitive functions are key elements of this cascade [13]. These pathological alterations are accompanied by regional glucose hypometabolism and progressive structural brain atrophy that develop over many years before the onset of overt dementia [14,15]. Importantly, these changes extend beyond medial temporal structures to distributed cortical networks, including the posterior cingulate and temporoparietal cortex, regions known to be particularly vulnerable in Alzheimer’s disease [16].

Important tools for describing these illness processes in vivo are provided by neuroimaging biomarkers. Cortical thinning and regional brain atrophy can be evaluated by structural magnetic resonance imaging (MRI), especially in medial temporal regions like the hippocampus and entorhinal cortex [17]. Fluorodeoxyglucose positron emission tomography (FDG-PET), which assesses neuronal metabolic activity, typically reveals hypometabolism in the temporoparietal and posterior cingulate regions in individuals with Alzheimer’s disease dementia [14,15]. Combining structural and metabolic imaging markers may offer a more complete view of disease processes than single-modality techniques alone since these modalities capture complimentary features of neurodegeneration [3].

Alongside advancements in biomarker research, the use of deep learning (DL), machine learning (ML), and artificial intelligence (AI) approaches has increased in Alzheimer’s disease research. Across the AD spectrum, these computational techniques have been applied to automated diagnosis, disease severity prediction, and progression risk estimation [18]. When compared to single-modality models, some studies have shown that multimodal integration of imaging variables, especially combinations of structural MRI and PET biomarkers, can improve prediction performance [19,20]. In addition to highlighting the potential of multimodal biomarker integration and highlighting the rapid spread of AI-based techniques in AD diagnosis and prognosis, recent high-impact studies have also identified important methodological issues that still need to be addressed [12].

The current machine learning literature is still methodologically diverse and dispersed despite these developments. Preprocessing pipelines, model architectures, evaluation techniques, and input modalities vary significantly amongst research [19]. Concerns about generalizability across various clinical populations and imaging protocols are raised by the fact that many models are created and assessed largely using a small number of research cohorts, most notably the Alzheimer’s Disease Neuroimaging Initiative (ADNI) [20,21]. In addition, risks of bias such as data leakage, suboptimal validation design, and overly optimistic performance reporting have been widely documented in clinical AI research [22]. In order to achieve accurate and impartial estimations of model performance, methodological studies have highlighted the significance of rigorous validation procedures, such as nested cross-validation and strictly regulated preprocessing pipelines [23,24,25].

It is still challenging to transform these models into practical therapy tools, despite the rapid development of machine learning applications for Alzheimer’s disease. Numerous existing approaches rely on small study cohorts, specific input modalities, or simplified validation processes, which may limit generalizability and make practical implementation difficult. Experimental designs that specifically take into consideration methodological bias, multimodal biological complexity, and the variety of memory-clinic groups are necessary to address these shortcomings. Here, we investigate whether integrating multimodal neuroimaging and clinical data within a rigorously controlled machine learning framework can improve risk stratification across the Alzheimer’s disease continuum. To minimize bias and ensure reliable performance estimation, the modeling pipeline incorporates nested cross-validation in which preprocessing, feature selection, and hyperparameter optimization are fully embedded within the validation structure [23]. Validation is conducted at the participant level to prevent information leakage across repeated visits and to better reflect clinically realistic prediction scenarios. By leveraging real-world multimodal data and leakage-resistant validation, this study aims to bridge the gap between methodological machine learning research and clinically meaningful risk stratification in heterogeneous memory clinic populations, conceptualizing Alzheimer’s disease progression as a heterogeneous risk spectrum rather than a binary classification problem.

## 2. Materials and Methods

### 2.1. Study Design and Participants

This retrospective study utilized real-world data derived from a tertiary memory clinic population. Participants were classified as having either mild cognitive impairment (MCI) or Alzheimer’s disease (AD) according to standard National Institute on Aging–Alzheimer’s Association (NIA-AA) diagnostic criteria. MCI diagnoses were based on clinical and cognitive criteria, with biomarker information considered when available as part of routine clinical care. AD diagnoses were established in accordance with NIA-AA criteria and supported by Alzheimer’s disease-consistent PET findings. PET imaging was not systematically required for diagnostic confirmation, and clinical diagnoses were established by experienced clinicians based on comprehensive evaluation including clinical examination, cognitive testing, and available imaging findings.

Structural magnetic resonance imaging (MRI) data were processed using FreeSurfer (version 7.3.2; http://surfer.nmr.mgh.harvard.edu/) [26,27]. Automated cortical reconstruction and volumetric segmentation were performed using the standard recon-all pipeline. Regional volumetric measures, including bilateral hippocampal volumes and additional subcortical structures, were extracted. To account for inter-individual differences in head size, volumetric measures were normalized by estimated total intracranial volume (eTIV). Quality control was conducted through visual inspection of segmentation outputs, and cases exhibiting major segmentation errors were excluded from further analysis. The resulting volumetric features were subsequently incorporated into the multimodal modeling framework.

FDG-PET images were acquired as part of routine clinical care. PET images were spatially normalized and co-registered to each participant’s structural MRI. Regional cerebral glucose metabolism was quantified using an atlas-based region-of-interest approach. Standardized uptake value ratios (SUVrs) were calculated by normalizing regional uptake values to the cerebellar cortex, which served as the reference region. PET-derived regional metabolic features were then incorporated into the multimodal machine learning framework. Baseline demographic and clinical characteristics of the cohort, including age, sex, education, and relevant clinical parameters, were compared between the MCI and AD groups using appropriate statistical tests. Potential confounding variables, particularly age and sex, were evaluated and considered among candidate model features where appropriate.

All diagnoses were established by qualified medical specialists based on comprehensive clinical evaluation, including neuroimaging findings and cognitive testing. The primary objective of the study was to develop and evaluate multimodal machine learning models capable of distinguishing between AD and MCI, and to examine whether these models could be applied to risk enrichment within the MCI population.

### 2.2. Multimodal Data Acquisition

**Figure 1:**
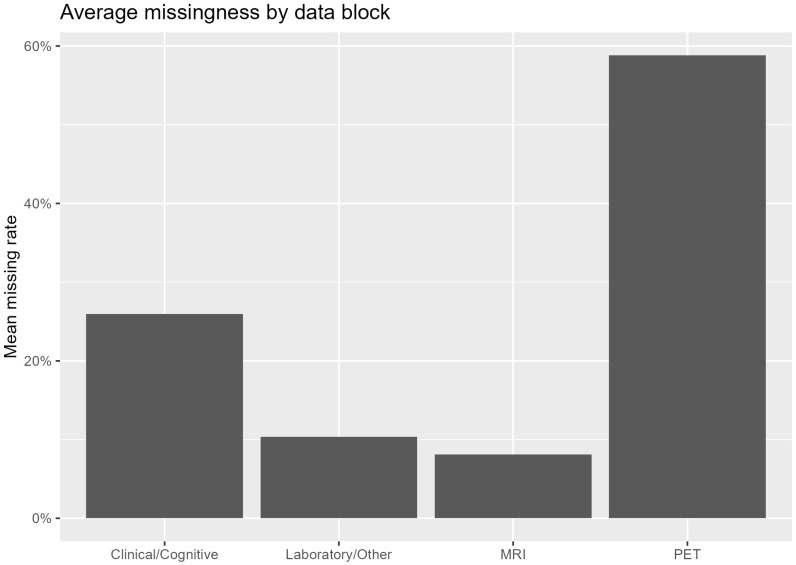
Average proportion of missing data across modality-specific feature blocks.

The dataset comprised clinical and demographic variables, laboratory parameters, neuropsychological test results, structural MRI-derived regional brain volumes, and PET-derived regional uptake measures. Given the constraints inherent to actual clinical practice, not all participants had data available for every modality, with advanced imaging modalities exhibiting substantial missingness (Figure 1; Supplementary Figure S1).

To ensure temporal consistency across modalities, clinical, laboratory, MRI, and PET data were aligned within a ±6-month window. Missingness rates shown in Figure 1 and Supplementary Figure S1 reflect patient-wise modality availability across the cohort, whereas multimodal data used for modeling were temporally aligned within a ±6-month window to ensure temporal consistency.

### 2.3. Handling of Missing Data and Modality Availability

Given the structured and non-random nature of missing data—particularly for PET imaging—no global imputation strategy was applied. Instead, analyses were conducted using modality-specific feature sets to reflect realistic diagnostic processes and real-world modality availability. Since the primary objective was to reflect real-world modality availability rather than improve performance under idealized assumptions, no formal sensitivity analyses utilizing other imputation procedures were performed (Figure 1, Supplementary Figure S1).

### 2.4. Feature Sets and Preprocessing

Feature sets were constructed incrementally, beginning with clinical and laboratory variables, followed by the addition of MRI-derived volumetric features and PET-derived regional uptake measures. Continuous variables were standardized prior to model training. All preprocessing steps, including feature scaling and any feature selection procedures, were performed exclusively within the training folds of the cross-validation framework.

### 2.5. Machine Learning Models

Extreme Gradient Boosting (XGBoost, version 1.7.6) and Elastic Net regularized logistic regression (scikit-learn, version 1.3) were used. XGBoost was selected to capture any non-linear correlations, and Elastic Net for its robustness and interpretability in high-dimensional, correlated data.

### 2.6. Cross-Validation Strategy

Model performance was evaluated using nested cross-validation performed strictly at the participant level to avoid information leakage. All visits from a given individual were assigned exclusively to either training or test folds. Hyperparameter optimization and feature selection were performed exclusively within the inner cross-validation loop, while the outer loop was used to estimate unbiased model performance. Nested cross-validation consisted of 5 outer folds and 5 inner folds.

### 2.7. Model Evaluation Metrics

**Figure 2:**
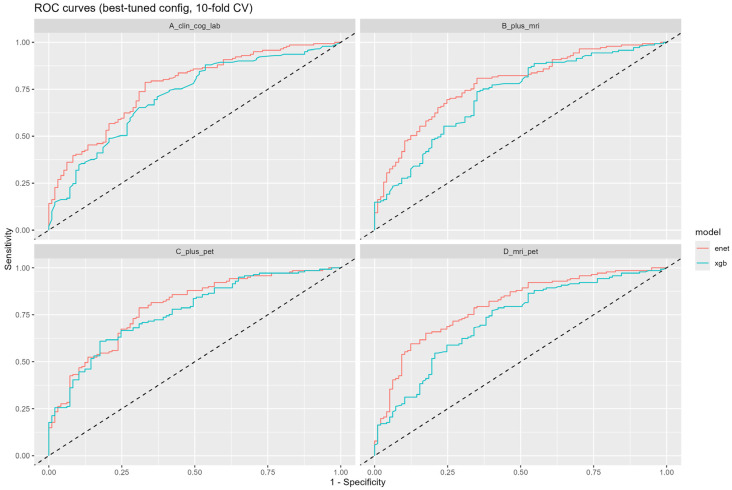
ROC curves across feature sets (10-fold cross-validation). The dashed diagonal line indicates the performance of a random classifier (AUC = 0.5).

Model discrimination was assessed using the area under the receiver operating characteristic curve (AUC). Ninety-five percent confidence intervals (95% CIs) were estimated based on variability across outer cross-validation folds. Given class imbalance, precision-recall curves were performed for additional evaluation (Figure 2, Supplementary Figure S5).

### 2.8. Risk Enrichment Analysis in MCI

**Figure 3:**
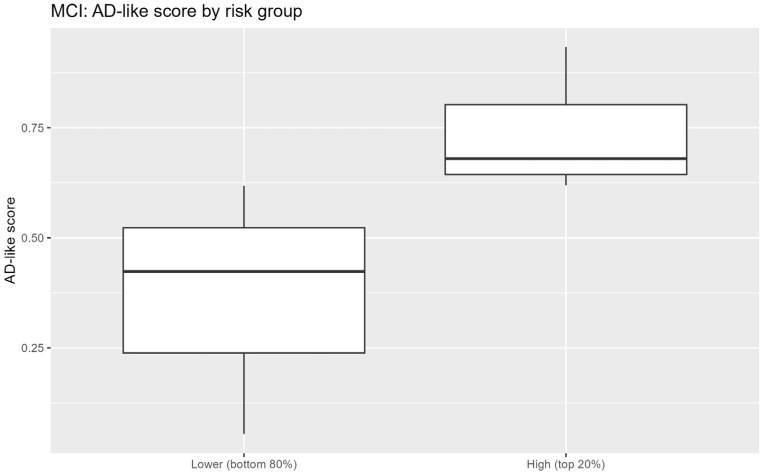
AD-like risk score stratification within the MCI cohort.

Within the MCI subgroup, continuous AD-like risk scores were generated using the best-performing multimodal model, which incorporated clinical, laboratory, MRI, and PET variables. For exploratory analyses, the top 20% of MCI patients were designated as a high-risk subgroup. This threshold was selected for pragmatic risk enrichment and exploratory purposes and rather than as a clinically validated cutoff (Figure 3).

### 2.9. Unsupervised Analyses

Principal component analysis (PCA) and hierarchical clustering were applied to standardized multimodal features to explore latent data structure and phenotypic continuity between MCI and AD. These unsupervised analyses were conducted solely for exploratory visualization and were not used for feature reduction or model training (Supplementary Figures S8–S10). Unsupervised analyses were performed on available complete-case subsets for the selected variables and were intended solely for exploratory visualization; no imputation was applied, and these analyses were not used for model training, feature selection, or performance evaluation.

## 3. Results

### 3.1. Cohort Characteristics and Data Availability

The final dataset comprised 1547 clinical visits from 474 distinct patients. Approximately 40% of patients had PET imaging available, whereas clinical and structural MRI data were more broadly available. Consistent with actual clinical workflows, missingness patterns indicated that advanced imaging modalities accounted for the majority of unavailable data (Figure 1; Supplementary Figure S1).

### 3.2. Group-Level Differences Between MCI and AD

**Figure 4:**
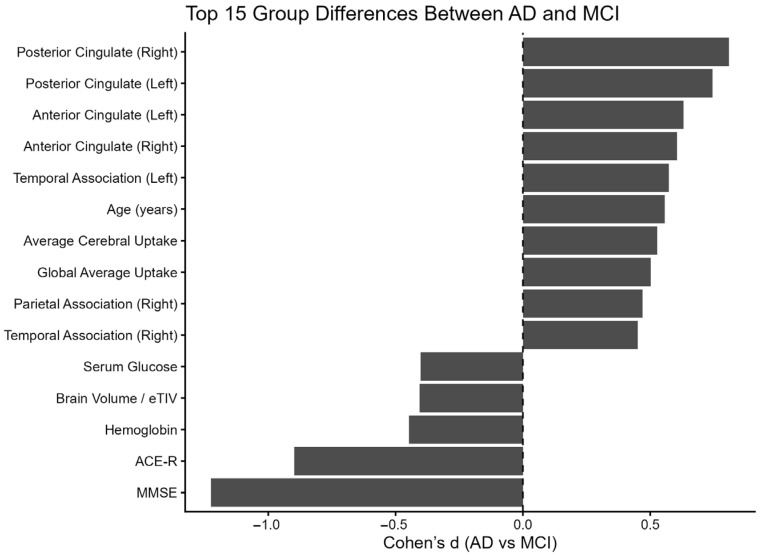
Top 15 group differences between AD and MCI (Cohen’s d).

Effect size analyses revealed significant differences between the MCI and AD groups, particularly in global cognitive assessments and PET-derived metabolic areas (Figure 4). The largest effect sizes were observed for global neuropsychological assessments, including the Mini-Mental State Examination (MMSE) and Addenbrooke’s Cognitive Examination—Revised (ACE-R) (Supplementary Figures S2 and S4). PET-derived metabolic metrics in posterior cingulate and temporoparietal regions demonstrated more pronounced group-level differences (Figure 4), whereas individual MRI volumetric measures, such as hippocampal volumes, exhibited substantial overlap between groups (Supplementary Figure S3).

### 3.3. Classification Performance Across Modalities

Model discrimination improved incrementally with the inclusion of additional data modalities. Models based solely on clinical and laboratory variables demonstrated moderate performance, while the inclusion of MRI-derived features yielded consistent improvements in AUC. The highest discriminative performance was achieved by multimodal models incorporating PET features. The multimodal model achieved an AUC of 0.87 (95% CI: 0.82–0.92), outperforming models based on clinical variables alone (AUC 0.74, 95% CI 0.68–0.80) and models including MRI features (AUC 0.80, 95% CI 0.75–0.85) (Figure 2). Elastic Net models exhibited stable performance across cross-validation folds, whereas XGBoost models showed greater variability. Precision–recall analyses further supported the potential utility of multimodal models for screening and risk enrichment purposes, rather than for definitive diagnosis (Supplementary Figure S5). Because PET imaging was available in a subset of participants, multimodal models incorporating PET were trained on the corresponding subset with available PET data, whereas unimodal models were evaluated using all available cases for the respective modality.

### 3.4. Feature Contributions

Regularized coefficients from Elastic Net models and permutation-based importance analyses consistently identified global cognitive measures and PET-derived regional uptake features as the most influential predictors (Supplementary Figures S6 and S7). MRI volumetric features contributed additional, albeit comparatively smaller, discriminative value.

### 3.5. Risk Enrichment Within the MCI Population

**Figure 5:**
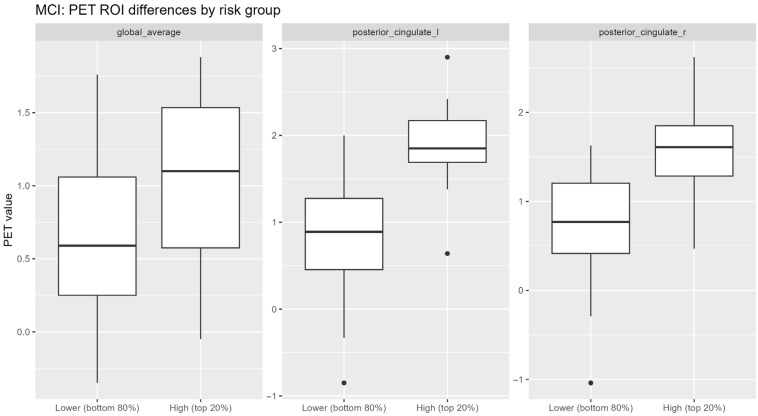
PET ROI differences between MCI risk groups.

Application of the best-performing multimodal model to the MCI subgroup yielded a continuous distribution of AD-like risk scores (Figure 3). Stratified analyses indicated that individuals within the highest-risk quintile exhibited greater PET-derived uptake abnormalities in AD-related regions, including the posterior cingulate cortex, compared with lower-risk groups (Figure 5). Despite partial overlap between risk strata, these findings are consistent with biological enrichment rather than with discrete subgroup separation.

### 3.6. Unsupervised Structure of Multimodal Data

Principal component analysis demonstrated that variance was distributed across multiple components, reflecting substantial high-dimensional heterogeneity (Supplementary Figure S8). Hierarchical clustering and silhouette analysis failed to identify well-separated groupings that matched clinical diagnosis (Supplementary Figure S9). The substantial overlap between AD and MCI instances in the PCA space supported a continuum-based model (Supplementary Figure S10).

## 4. Discussion

This study demonstrates that multimodal machine learning models integrating routinely acquired clinical measures with neuroimaging, particularly FDG-PET, can discriminate Alzheimer’s disease (AD) from mild cognitive impairment (MCI) and generate continuous AD-like risk scores that meaningfully enrich for biologically vulnerable individuals within the MCI spectrum. Across analyses, PET-derived metabolic abnormalities in posterior default mode network regions, including the posterior cingulate and temporoparietal cortex, emerged as the most influential contributors, whereas structural MRI measures showed substantial overlap at the individual level. These results are in line with evidence that metabolic changes may occur before or beyond observable structural atrophy in the early stages of the disease, as well as the well-established vulnerability of posterior default mode network hubs in Alzheimer’s disease [28,29,30].

This pattern is biologically plausible and consistent with a large body of research showing that one of the earliest and most frequently reported functional indicators of Alzheimer’s disease is hypometabolism in the posterior cingulate and precuneus, which can be detected even before overt dementia or significant structural atrophy [28,29]. Crucially, convergent data suggest that metabolic dysfunction reflects early synaptic and network-level failure within large-scale networks such as the default mode network, which may precede or only partially overlap with macroscopic neuronal loss, particularly during prodromal stages [30,31,32]. Although hippocampal volume and other structural markers remain biologically significant indicators of neurodegeneration, inter-individual variability and stage-dependent effects constrain their ability to discriminate, highlighting the importance of complementary modalities rather than depending solely on one biomarker [33,34]. Taken as a whole, these findings provide a biological rationale for multimodal integration when evaluating risk and disease expression across the MCI–AD spectrum, supporting a continuum-based perspective in which metabolic, structural, and cognitive alterations provide different but connected information [35,36].

From a methodological perspective, our findings align with and expand prior work demonstrating that multimodal machine learning approaches integrating PET with structural imaging and clinical data can improve discrimination along the Alzheimer’s disease continuum when supported by rigorous experimental design and validation strategies. Several studies have shown that FDG-PET contributes disproportionately to classification performance, particularly for differentiating prodromal stages, due to its sensitivity to early network-level dysfunction within large-scale cortical systems such as the default mode network [37,38]. However, methodological reviews consistently highlight that reported performance gains are heavily dependent on feature selection procedures, validation design, and handling of modality-specific missingness, with inadequate approaches leading to systematic overestimation of model accuracy [19]. In this context, PET should be viewed not merely as a performance-enhancing input, but as a biologically distinct modality whose contribution must be evaluated within leakage-resistant, patient-level validation frameworks, ensuring that model performance reflects genuine biological signal rather than methodological artefacts. This methodological perspective also provides an important context for interpreting recent low-dimensional machine learning models that aim to balance predictive performance with clinical interpretability.

In parallel with multimodal approaches, several recent studies have proposed low-dimensional machine learning frameworks aimed at improving clinical interpretability while maintaining competitive predictive performance. For example, Christodoulou and colleagues developed ensemble-based classifiers using compact feature sets derived from clinical, cognitive, and imaging variables, incorporating explicit uncertainty handling and decision deferral mechanisms to improve transparency in clinical decision support [39,40]. These approaches demonstrate that interpretable models using a limited number of well-defined predictors can achieve competitive diagnostic performance while facilitating clinical trust and model explainability. However, their reliance on relatively restricted feature spaces may limit the ability to capture the full biological heterogeneity of the Alzheimer’s disease continuum. In contrast, our multimodal framework emphasizes biologically grounded integration of metabolic, structural, and clinical information while maintaining stringent validation procedures designed to reduce overfitting and methodological bias [12]. Importantly, whereas many low-dimensional models prioritize interpretability through aggressive feature reduction, such simplification may risk underrepresenting the biological complexity of the Alzheimer’s disease continuum, particularly when metabolic and structural imaging signals provide complementary information across distributed neural systems.

A growing body of evidence indicates that PET-based biomarkers—particularly FDG-PET—capture biologically meaningful aspects of Alzheimer’s disease that do not necessarily align with categorical clinical diagnoses, but instead reflect underlying neurodegenerative burden and disease activity [41,42,43]. Hypometabolism within posterior default mode network hubs—especially the posterior cingulate cortex and precuneus—emerges as a robust and reproducible signal across heterogeneous MCI and AD populations, despite substantial inter-individual variability in regional patterns [43,44]. Importantly, several studies report that FDG-PET abnormalities are more closely associated with near-term cognitive decline than amyloid burden alone, particularly among amyloid-positive individuals, supporting the role of metabolic measures as indicators of active disease processes rather than static pathology [10,14].

Explainable and modality-aware modeling approaches further suggest that the value of PET lies not only in improving discrimination, but also in providing interpretable links between metabolic dysfunction and disease stage, complementing structural markers that are more strongly influenced by inter-individual variability and stage-dependent effects [45]. By adopting a conservative multimodal strategy that incrementally integrates PET features, explicitly accounts for real-world modality availability, and prioritizes robust validation over maximal accuracy, our approach reflects an emerging methodological consensus: clinically meaningful multimodal machine learning models must balance biological plausibility, interpretability, and generalizability rather than optimizing predictive performance in isolation [19,37].

This dissociation between clinical labels and biomarker expression should not necessarily be interpreted as a methodological limitation, but rather reflects the heterogeneous and temporally dynamic nature of Alzheimer’s disease biology [41]. Multimodal imaging studies further demonstrate that integrating metabolic, molecular, and structural information improves biological interpretability and supports the use of probabilistic—rather than deterministic—frameworks when linking biomarkers to clinical outcomes [15,44]. Consistent with this literature, our findings suggest that the AD-like risk scores derived from multimodal models capture biologically plausible metabolic patterns, particularly within posterior default mode network hubs centered on the posterior cingulate cortex and precuneus. Importantly, these scores should be interpreted as indicators of biological vulnerability rather than as definitive diagnostic or treatment decision thresholds, aligning with biological validation frameworks that prioritize sensitivity to disease-related processes over strict categorical classification [46,47].

Growing evidence indicates that heterogeneity within the Alzheimer’s disease spectrum—particularly at the MCI stage—substantially reduces statistical power and obscures treatment effects in both observational studies and clinical trials, often leading to inconclusive or falsely negative results despite biologically active interventions [2,48]. In this context, risk enrichment has emerged not merely as a diagnostic refinement, but as a conceptual framework aimed at identifying individuals more likely to exhibit clinically meaningful progression within a relevant time window. Such approaches are intended to improve trial efficiency and interpretability rather than diagnostic accuracy per se [2,48]. Importantly, amyloid positivity alone has been shown to be insufficient for short- to medium-term prognostic stratification, underscoring the need for complementary biological dimensions—such as metabolic and neurodegenerative markers—that are more closely aligned with downstream clinical change [2]. Recent work further emphasizes that enrichment strategies should embrace, rather than suppress, biological and phenotypic heterogeneity by favoring continuous, distribution-based risk estimates over fixed diagnostic thresholds, which are poorly suited to the dynamic and multifactorial nature of Alzheimer’s disease [49,50].

In real-world memory clinic settings, where missingness and variability are inherent, risk enrichment has been proposed as an interpretative layer that enhances clinical relevance without inflating model performance claims or conflating prediction with diagnosis [51,52]. Critically, several authors caution that risk scores should be positioned as decision-support tools rather than diagnostic surrogates, a distinction with both methodological and ethical implications, particularly in the context of emerging disease-modifying therapies [53,54]. From a trial-design perspective, multimodal enrichment strategies integrating imaging and other biological markers have been shown to increase statistical power and reduce required sample sizes, reinforcing the role of risk-based stratification as a prospective design strategy rather than a post hoc analytic convenience [55].

Taken together, these conceptual and empirical advances support the framing of the present model outputs as biologically informed risk gradients that align with the continuum view of Alzheimer’s disease, offering a pragmatic enrichment layer compatible with both real-world clinical practice and contemporary research settings. Importantly, the interpretation of risk enrichment signals must be situated within the substantial biological heterogeneity that characterizes the MCI–AD spectrum [56,57,58]. Growing evidence suggests that heterogeneity is not a source of analytical noise, but a defining feature of the MCI–AD spectrum [57].

Data-driven and rule-based techniques have demonstrated that individuals sharing the same clinical diagnosis may exhibit markedly different biological and clinical trajectories ranging from rapid progression to long-term stability or even reversion. These differences reflect underlying diversity in cognitive profiles, network involvement, and biological substrates [57,59]. This heterogeneity emphasizes that prognostic variability is intrinsically temporal and cannot be adequately captured by static diagnostic labels alone, since it extends beyond baseline phenotypes to longitudinal illness histories [58,59]. Multimodal studies further demonstrate that similar levels of cognitive impairment may arise from distinct neuroanatomical, metabolic, and network-level patterns, supporting the view that Alzheimer’s disease progresses through multiple, partially overlapping biological pathways rather than a single linear route [49,60,61].

Importantly, observations from real-world cohorts highlight that systemic factors and comorbidities contribute additional layers of heterogeneity, reinforcing the need for integrative models that move beyond isolated biomarkers and acknowledge the multidimensional nature of disease expression [62]. In this context, the absence of sharply delineated clusters and the presence of overlapping risk distributions, such as those observed in our multimodal analyses and PCA space, are consistent with established biological evidence rather than indicative of methodological limitations. Together, these findings support framing Alzheimer’s disease as a continuum characterized by individual-specific trajectories, for which continuous, distribution-based risk measures offer a more biologically faithful and clinically meaningful representation than rigid categorical classifications [49,57,58].

Despite the growing body of work demonstrating high classification performance of multimodal machine learning models in Alzheimer’s disease, important barriers to clinical translation remain. Previous research has demonstrated that many reported performance gains are derived from controlled research cohorts, most notably ADNI, and may therefore overestimate real-world generalizability, especially in the presence of longitudinal variability, missing data, and heterogeneous patient profiles [63,64]. Methodological issues such as data leakage, insufficient validation techniques, and overfitting further limit interpretability and reliability, reinforcing the necessity of patient-level validation and leakage-aware evaluation frameworks [20]. The importance of modality-aware and incremental modeling strategies that align with real-world diagnostic pathways is further highlighted by the fact that multimodal approaches combining MRI and PET often outperform single-modality approaches, while their reliance on costly or inconsistently available inputs constrains routine clinical deployment [13,65].

From a translational standpoint, a growing body of evidence suggests that ML-derived outputs and imaging biomarkers are better positioned as decision-support tools rather than final diagnostic classifiers, helping to reduce clinical uncertainty and guide the targeted use of advanced investigations in selected patients [15,66]. Explainability and model transparency are equally critical because black-box technologies lacking interpretable outcomes are unlikely to gain clinician trust or ethical acceptance in routine practice [67].

Finally, the value of risk-based enrichment frameworks is further reinforced by practical and economic factors. Biologically informed stratification may support more effective use of emerging disease-modifying therapies and advanced imaging, as opposed to in-discriminate testing across heterogeneous populations [68]. In this context, the present study does not seek to replace clinical diagnosis or treatment decision-making, but rather to provide a biologically grounded, interpretable risk layer that reflects real-world complexity and may support more targeted, efficient, and clinically meaningful applications of multimodal biomarkers.

Several limitations should be considered when interpreting these findings. Firstly, this study was conducted retrospectively in a single-center memory clinic cohort, which may limit generalizability to other clinical populations and healthcare systems. Secondly, PET imaging was available only in a subset of participants, reflecting real-world diagnostic pathways but potentially introducing sampling differences across modality-specific analyses. Thirdly, although diagnoses were established by experienced clinicians using standard NIA–AA criteria based on comprehensive clinical and cognitive evaluation, PET imaging was available as part of routine clinical assessment and may have contributed to diagnostic adjudication in some cases. Consequently, the prominence of PET-derived features in the machine learning models should be interpreted with caution, as partial diagnostic circularity cannot be entirely excluded. Finally, external validation in independent cohorts and prospective longitudinal studies will be important to confirm the robustness, clinical applicability, and potential prognostic value of the proposed multimodal risk framework. In addition, model outputs were interpreted as relative biological similarity scores rather than calibrated probabilities; therefore, formal calibration analyses were not performed.

Taken together, these findings support a biologically informed multimodal framework in which machine learning-derived risk estimates complement clinical assessment by capturing heterogeneous disease processes across the Alzheimer’s disease continuum. Future prospective validation in independent cohorts will be essential to determine whether such multimodal enrichment strategies can meaningfully improve clinical decision-making and facilitate more efficient trial design in Alzheimer’s disease.

## Data Availability

The data used in this study were obtained from a real-world tertiary memory clinic cohort and contain sensitive patient information. Due to ethical and privacy considerations, the datasets are not publicly available. Data may be made available from the corresponding author upon reasonable request, subject to institutional and ethical approval.

## Supplementary Materials

The following supporting information can be downloaded at: https://www.mdpi.com/article/10.3390/jcm15062250/s1.

## Abbreviations

ACE-R	Addenbrooke’s Cognitive Examination—Revised
AD	Alzheimer’s disease
AI	Artificial intelligence
AUC	Area under the receiver operating characteristic curve
DMN	Default mode network
EAN	European Academy of Neurology
eTIV	Estimated total intracranial volume
EN	Elastic Net
FDG-PET	Fluorodeoxyglucose positron emission tomography
MCI	Mild cognitive impairment
ML	Machine learning
MMSE	Mini-Mental State Examination
MRI	Magnetic resonance imaging
NIA-AA	National Institute on Aging–Alzheimer’s Association
PCA	Principal component analysis
PCC	Posterior cingulate cortex
PET	Positron emission tomography
PR	Precision–recall
ROC	Receiver operating characteristic
ROI	Region of interest
SUVr	Standardized uptake value ratio
XGBoost	Extreme Gradient Boosting
