# Supplementary material for: Real-World Multimodal Machine Learning for Risk Enrichment Across the Alzheimer’s Disease Spectrum
Source: J Clin Med. 2026 Mar 16;15(6):2250. doi: 10.3390/jcm15062250 (PMC13026771; doi:10.3390/jcm15062250)
Supplement: Supplementary file 1 [file jcm-15-02250-s001.zip › jcm-4138527-supplementary.pdf]

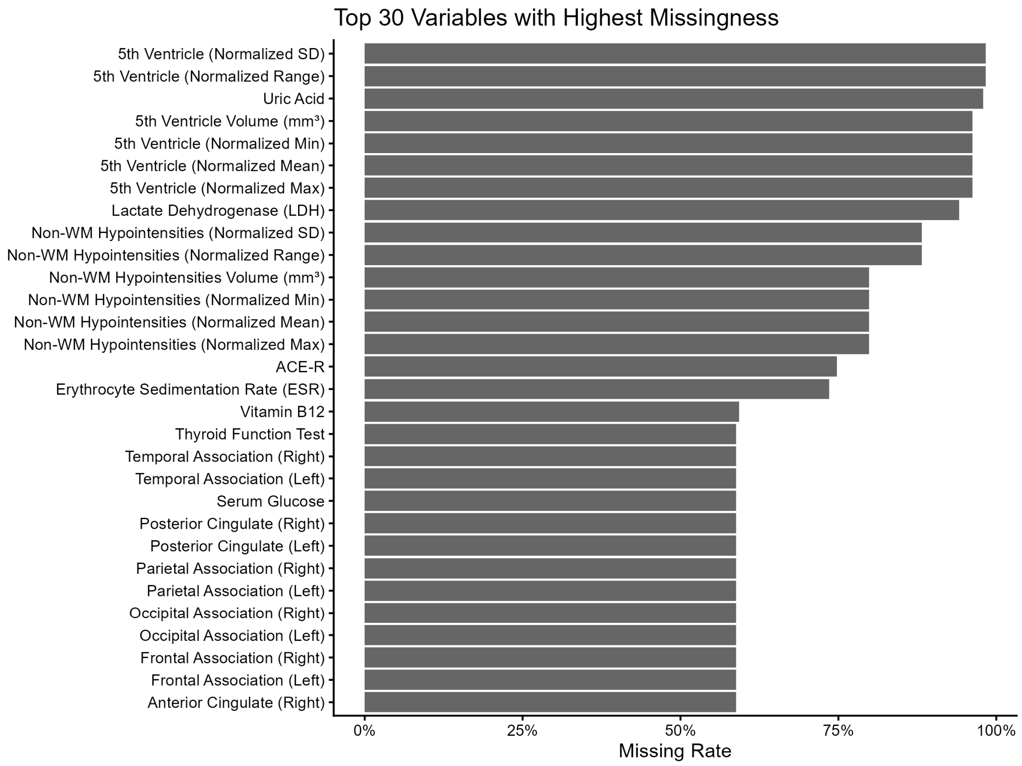

Supplementary Figure S1. Top 30 variables with the highest missingness

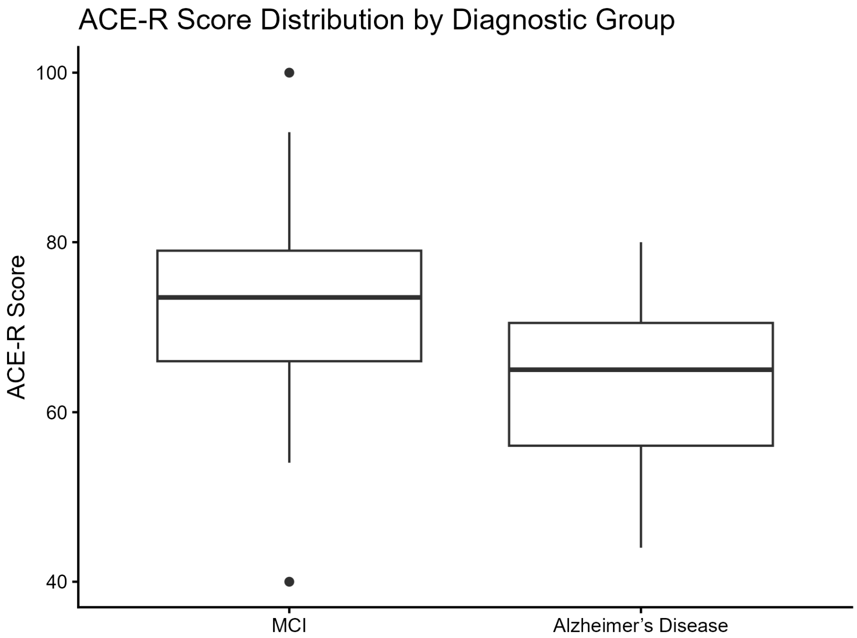

Supplementary Figure S2. ACE-R score distributions by diagnostic group (AD vs MCI)

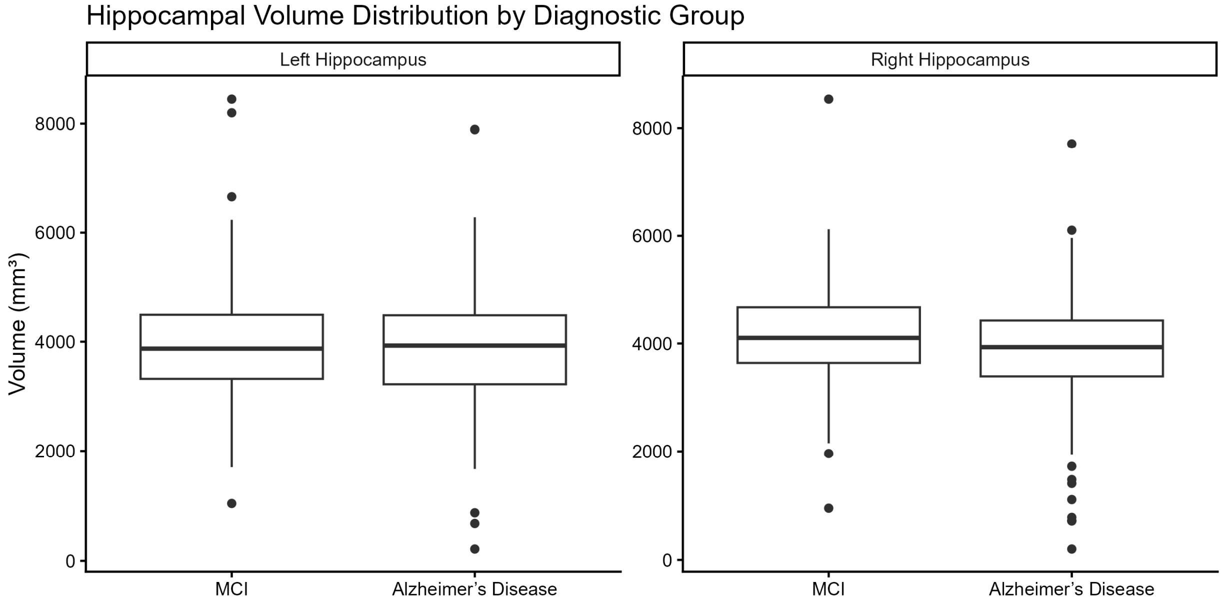

Supplementary Figure S3. Left and right hippocampal volume distributions by group

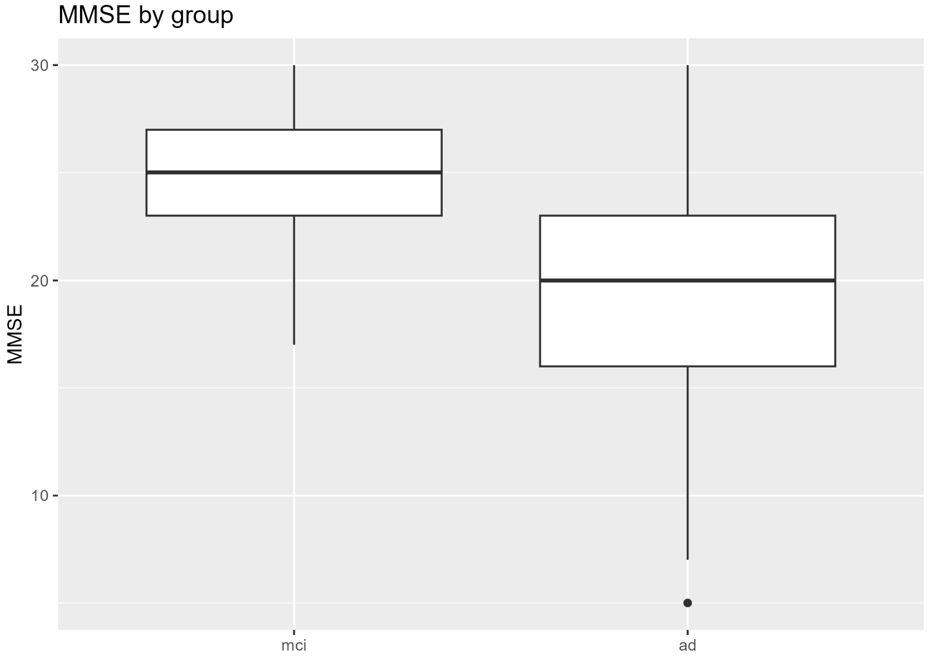

Supplementary Figure S4. MMSE score distributions by diagnostic group

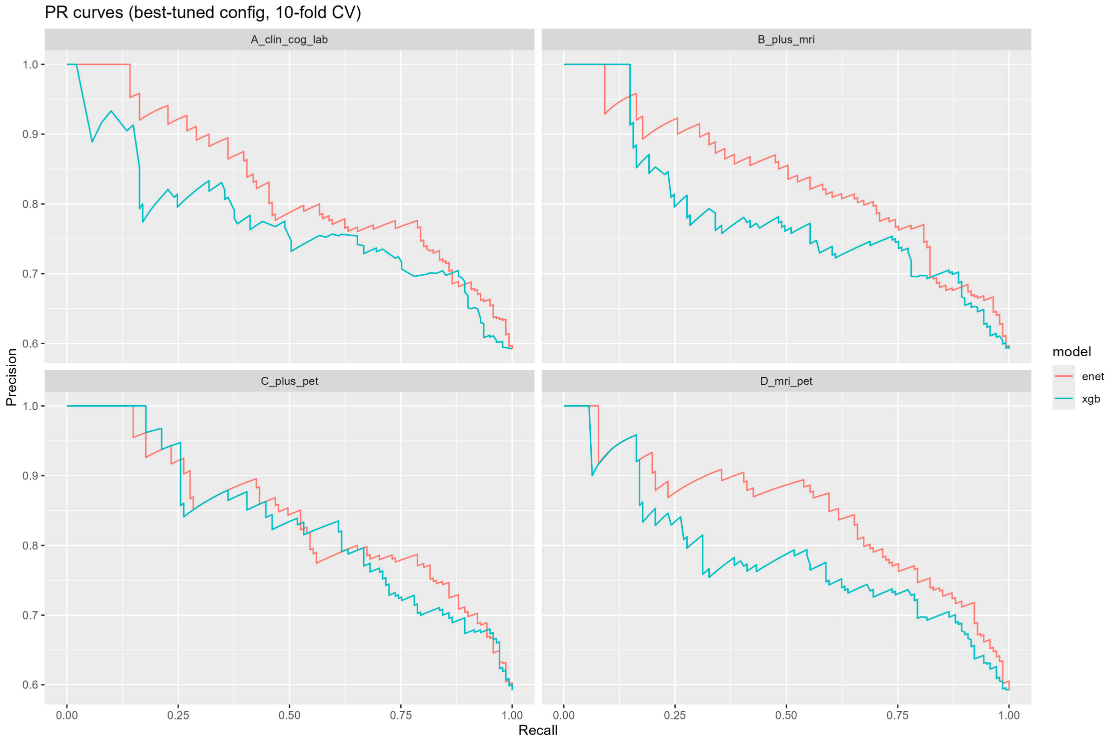

Supplementary Figure S5. Precision–recall curves across feature sets

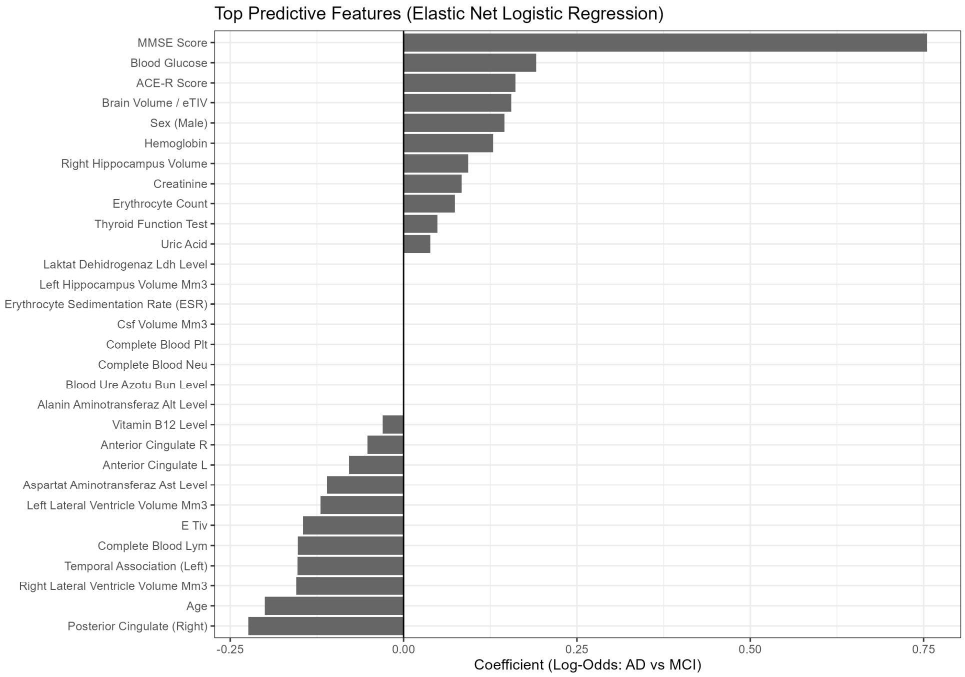

Supplementary Figure S6. Elastic Net logistic regression coefficients (top 30 features)

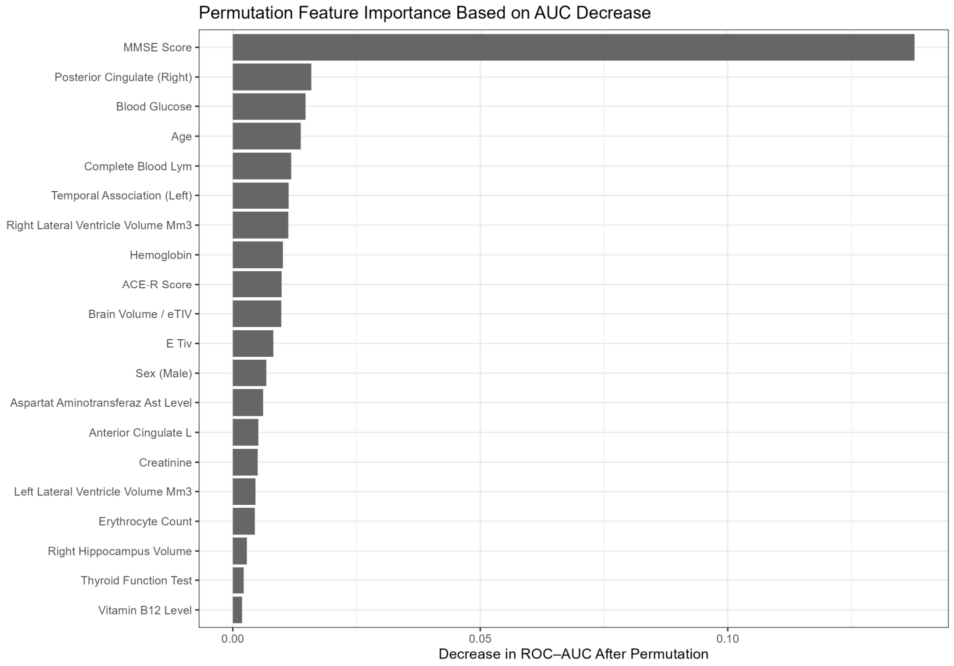

Supplementary Figure S7. Permutation-based feature importance (AUC drop analysis)

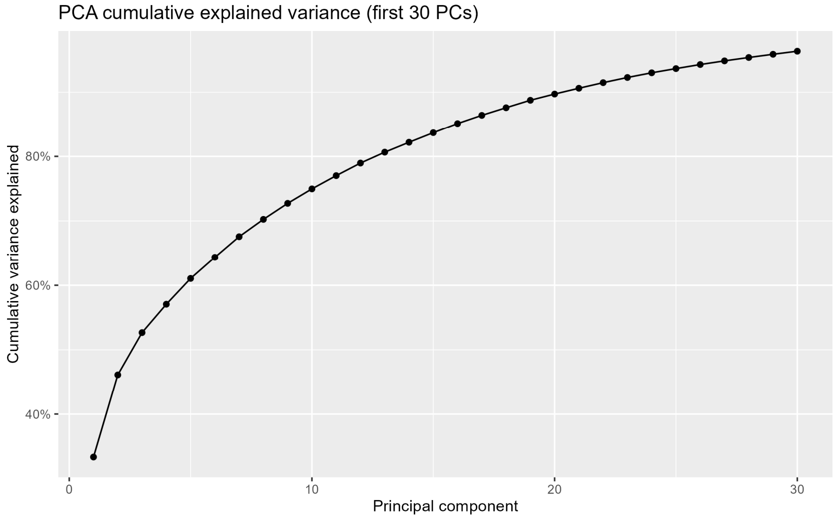

Supplementary Figure S8. Cumulative explained variance from PCA (first 30 principal components)

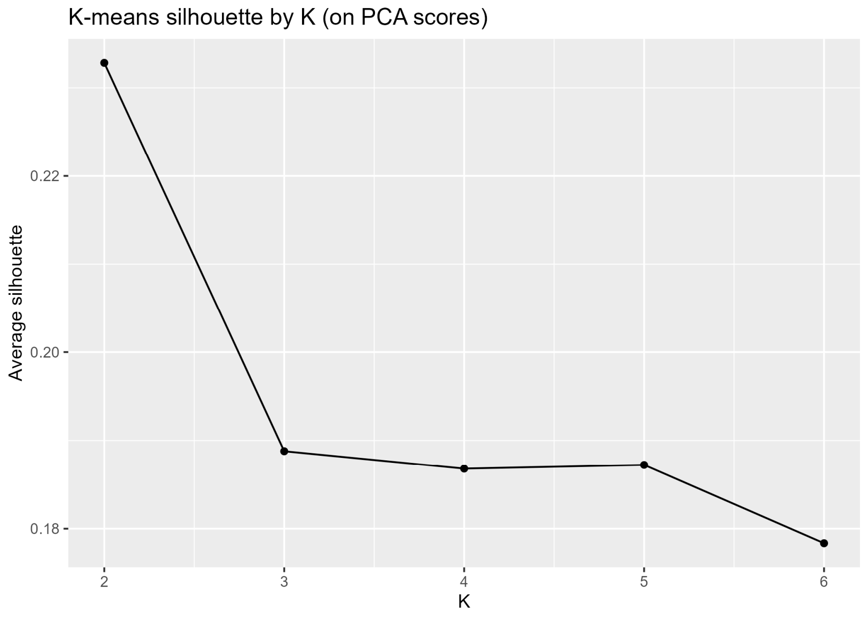

Supplementary Figure S9. Silhouette analysis for different cluster numbers (K)

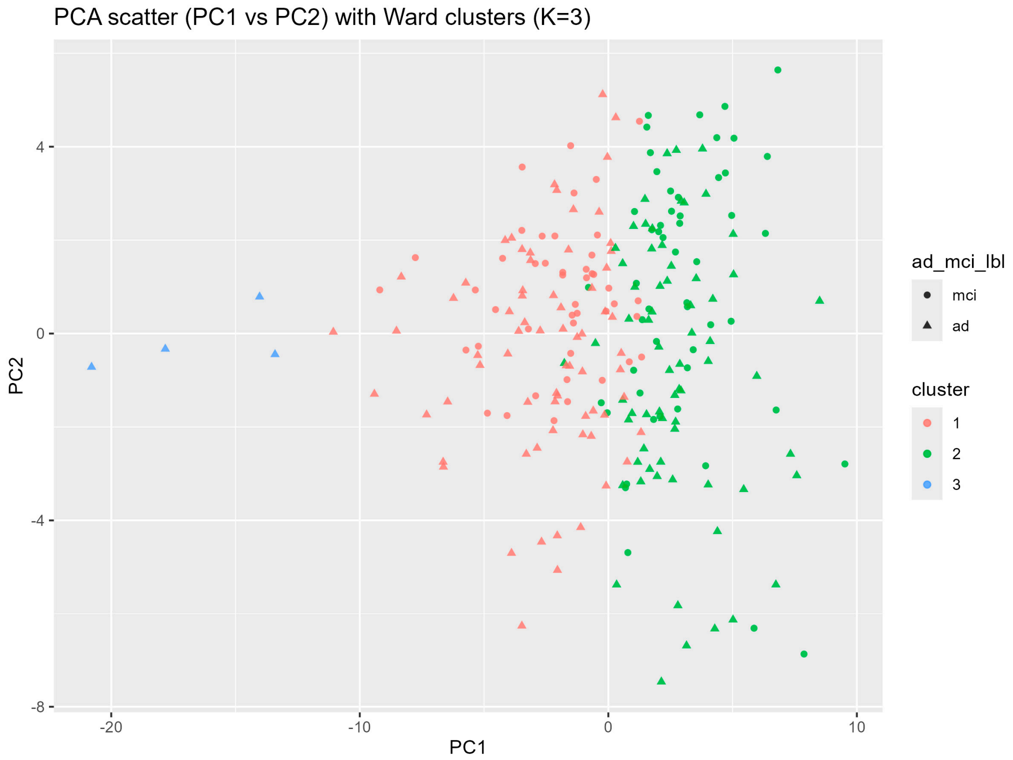

Supplementary Figure S10. PCA scatter plot (PC1 vs PC2) with Ward hierarchical clustering (K = 3)
